# Visualization of radiocesium distribution in surface layer of seafloor around Fukushima Daiichi Nuclear Power Plant

**DOI:** 10.1038/s41598-021-02646-9

**Published:** 2021-11-30

**Authors:** Yukihisa Sanada, Yoshimi Urabe, Toshiharu Misonou, Takehiko Shiribiki, Takahiro Nakanishi, Yusuke Watanabe, Tadahiko Tsuruta

**Affiliations:** 1 Sector of Fukushima Research and Development, Japan Atomic Energy Agency, 45-169 Sukakeba, Minamisoma, Fukushima 975-0036 Japan; 2 NESI Inc, 38 Shinko-cho, Hitachinaka, Ibaraki 312-0005 Japan; 3 Sanyo Techno Marine, 1-3-17 Horidomecho, Nihonbashi, Chuouku, Tokyo, 103-0012 Japan

**Keywords:** Environmental sciences, Physical oceanography

## Abstract

Large quantities of volatile radionuclides were released into the atmosphere and the hydrosphere following the Fukushima Daiichi Nuclear Power Plant (FDNPP) accident on March, 2011. Monitoring of radiocesium in sediment is important for evaluating the behavior of radiocesium in the environment and its effect on aquatic organisms. In this study, the radiocesium distribution in the surface sediment around the FDNPP was visualized as a radiocesium concentration map using periodical survey data from a towed gamma-ray detection system. The uncertainty of the radiocesium map was evaluated via comparison with a large amount of sediment core sample data. The characteristics of the radiocesium distribution were examined considering the seafloor topography and a geological map, which were obtained via acoustic wave survey. The characteristics of the formation of ^137^Cs anomaly at the estuaries were analyzed using a contour map of ^137^Cs concentration combined with water depth. Validation of the created map showed that it was comparable with actual sediment core samples. The map generated using the towed radiation survey depicted the ^137^Cs concentration distribution as the position resolution of a 1 km mesh. Finally, the ^137^Cs concentration decreased with time in consideration of such uncertainty.

## Introduction

Large quantities of volatile radionuclides were released into the atmosphere and the hydrosphere following the March, 2011 accident at the Fukushima Daiichi Nuclear Power Plant (FDNPP), which belongs to Tokyo Electric Power Company Holdings Inc.^1^. The terrestrial deposition of these radionuclides has been verified through continuous radiation survey^2,3^. Regarding radiocesium (^134^Cs and ^137^Cs), which has a relatively long half-life, certain national institutes are monitoring the seawater and bottom sediment off the coast of Fukushima to assess the radiocesium situation^4^. The concentration of radiocesium in the seawater has decreased since the accident ten years ago^5^. Radiocesium in sediment should be monitored to evaluate the behavior of radiocesium in the environment and its effect on aquatic organisms. Visualization of its distribution in coastal areas will provide information about the behavior mechanisms of environmental contaminants by interpreting radiocesium as an environmental tracer.

The total ^137^Cs released from the FDNPP was 15 to 18 PBq^5^. Approximately 1–2% of the ^137^Cs in the sea (0.2 ± 0.05 PBq) was deposited onto the sediment^6^. There is a larger amount of deposited radiocesium in seabed sediment near river mouths than that in pelagic areas, and radiocesium is distributed deeply throughout the seafloor^7^. Otosaka (2017) estimated that > 43% of ^137^Cs in the surface sediment around the FDNPP was transported to deeper sediment layers via vertical mixing of sediments from March 2011 to the end of 2015^8^. Moreover, radiocesium, which flows from rivers to seas, increases during flooding events, such as typhoons^9^. These evaluation values are based on limited monitoring data, such as grab sampling and short core sampling data of several dozens. These conventional methods of sediment sampling are insufficient for understanding the distribution characteristics of ^137^Cs off the coast of Fukushima.

A towed gamma-ray detection system is useful for determining the radiocesium distribution in surface sediment. Since the late 1950s, systems have been developed in at least ten countries, with a main breakthrough, such as technological advance of the battery and downsizing of the electronic circuit, emerging in the 1970s^10^. In the early 1980s, a smaller system was developed by the British Geological Survey for operation in small ships in nearshore waters^11^. This system was applied to surveys around the Sellafield Nuclear Fuel Reprocessing Plant to measure the distribution of radionuclides discharged from the plant into the nearshore seafloor sediment. Before the FDNPP accident, the Japan Atomic Energy Research Institute developed a submersible gamma-ray system that comprises a germanium detector mounted on a remotely operated vehicle^12^.

In November 2012, radiation survey of the surface sediment off the coast of Fukushima using a towed gamma-ray detection system started as a national project^13^. This system consisted of a waterproof scintillation detector and weight wrapped in an 8 m rubber hose. Periodical survey conducted approximately 10 km off the coast of the FDNPP revealed that radiocesium was accumulated in an area under a cliff. This phenomenon, which is based on the behavior of fine particles, is defined as the formation of radiocesium anomaly. This system was calibrated using simulation results and laboratory experiments^14^. Afterward, five survey campaigns were performed off the Fukushima coast in FY 2015–2018. However, these data have not been utilized officially because uncertainty evaluation via comparison with actual samples has been insufficient. Visualization of the radiocesium distribution in surface sediment using such detailed survey data is useful for the assessment of restarting the fishing industry. Moreover, it will lead to understanding the behavior of pollutants, such as the place where pollutants tend to accumulate, by comparison with visualized radiocesium maps and seafloor topography. In addition, to compare with different time data will lead to revealing the temporal change characteristics of radiocesium distribution.

In this study, the radiocesium distribution in the surface sediment around the FDNPP was visualized as a radiocesium map using periodical survey data from a towed gamma-ray detection system. The uncertainty of the radiocesium map was evaluated via comparison with large amounts of sediment core sample data. The characteristics of the radiocesium distribution were examined considering the seafloor topography and the geological map, which were obtained through an acoustic wave survey.

## Results and discussion

### Characteristics of ^137^Cs distribution

In this study, a towed radiation survey was conducted five times off the coast of Fukushima (approximately 50 km from north to south from the FDNPP and 20 km from the east of the FDNPP). The survey period is shown in Table 1. Two ^137^Cs contour maps were created: one by interpolation (mesh size: 250 m × 250 m) and one by mesh averaging (mesh size: 1 km × 1 km). The difference between the two maps was the position resolution and the corresponding accuracy. These differences are described in detail in the next section, and the characteristics of ^137^Cs distribution related to the seafloor topography are described on the basis of the interpolation map due to the high position resolution.

**Table 1:** Achievements of the survey campaign.

Campaign	Detector of towed radiation survey	Number of sediment core sample (for validation)	Start date	End date (corrected date)
1st	Small detector	34	16 December 2013	5 March 2014
2nd	Small detector + Large detector	59	14 November 2014	8 February 2015
3rd	Small detector + Large detector	47	30 September 2015	2 December 2015
4th	Large detector	42	21 November 2017	22 February 2018
5th	Large detector	21	27 July 2018	7 September 2018
Calibration	Large detector	150*	6 August 2020	20 August 2020

*Grab sampling.

**Figure 1:**
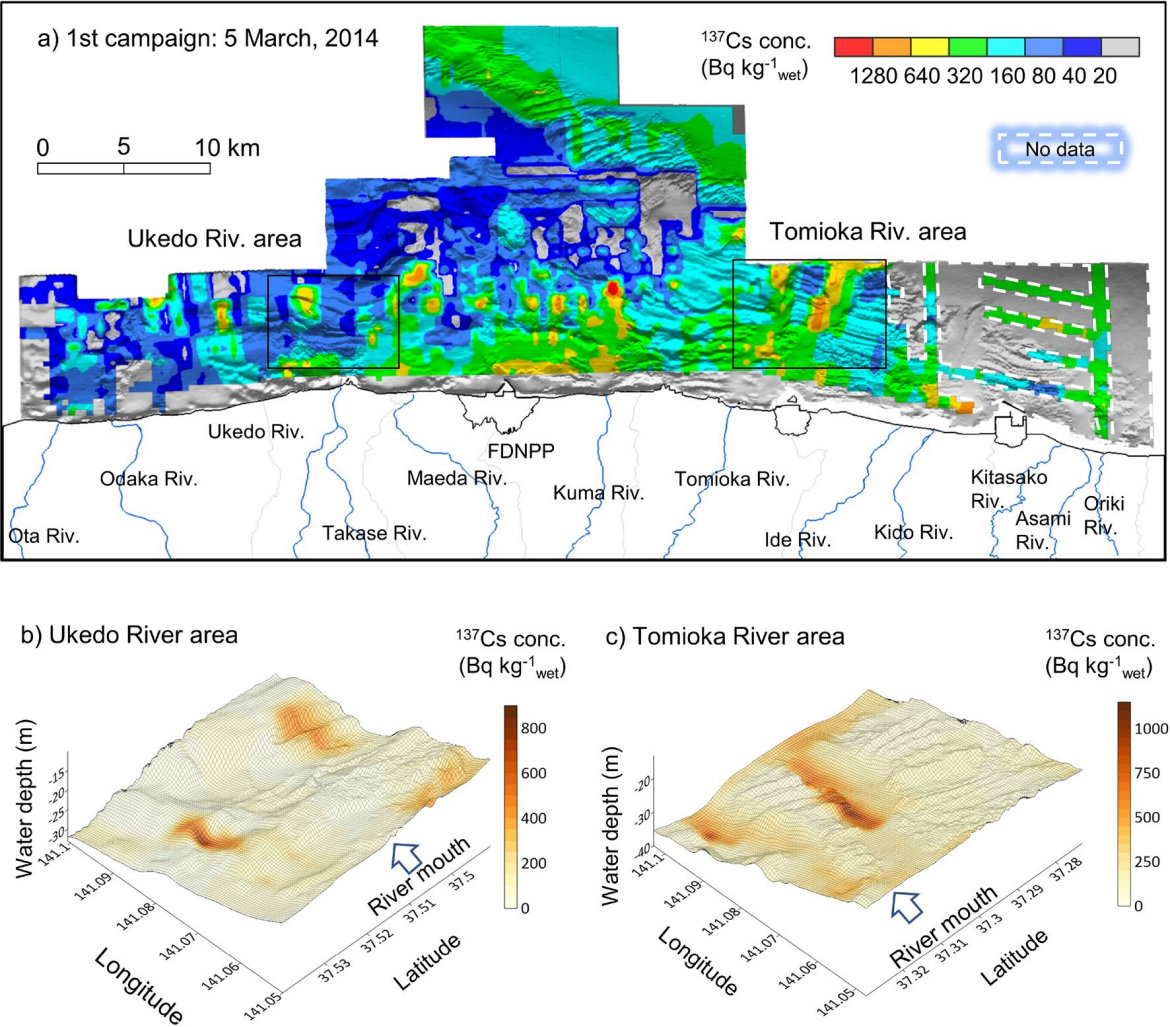
Contour map of ^137^Cs distribution on the topography map. (**a**) All estuary area. The enlarged map at estuaries of (**b**) Ukedo River and (**c**) Tomioka River at the 1st campaign. Map created with ESRI ArcGIS 10.7.1. Basemap sources: bathymetric chart in this study.

The contour maps of the ^137^Cs distribution in the first campaign on the topography map are shown in Fig. 1. This map created with ESRI ArcGIS 10.7.1. (Environmental Systems Research Institute Inc., California, USA) and basemap is M7000 Digital Bathymetric Chart (Shapefile) published by Marine Information Research Center (Tokyo, Japan). The concentration of ^137^Cs is expressed by a color image of eight intervals divided into two; with 20 Bq kg^−1^_wet_ as the approximate detection limit (DL). These maps were created by interpolation of accumulated data in a 250 m mesh. Areas lacking data are delineated by the white dotted line. The ^137^Cs concentration south of the FDNPP in the first campaign was relatively higher than that in the north, as shown in Fig. 1a. The borderline extension from the river mouth had a relatively high concentration along with the topography. In particular, a maximum concentration exceeding 1000 Bq kg^−1^_wet_ was observed 5 km from the mouth of the Kuma River in the first campaign. Detailed situations of the Ukedo River and Tomioka River, which have larger catchment areas, are shown in Fig. 1b,c. The topography of the Ukedo River estuary features a cliff that is shorter than 5 m and faces the river. Two areas with relatively high concentrations (anomalies) were observed 5 km away from the river mouth. Both anomalies were formed in the hollows of cliffs. By contrast, the Tomioka River estuary has two flat topography areas, which are along the borderline extension from the river mouth. Anomalies were observed along a cliff in these flat areas, 5 km from the river mouth. Radiocesium is transported to sea in combination with fine particles smaller than 10 μm^9,15^. The distribution characteristics in the contour maps showed that such fine particles flowing along the rivers were carried by approximately 5 km from the river mouth and accumulated in the hollow of a cliff. Thornton et al. (2013) estimated the relationship between the anomaly and the seafloor topography based on the result of towed radiation survey^13^. Thus, the distribution of the ^137^Cs concentration at the estuary depends on the seafloor topography and the inflow from the river. The anomaly formation process was elucidated through 3D visualization of the ^137^Cs concentration.

**Figure 2:**
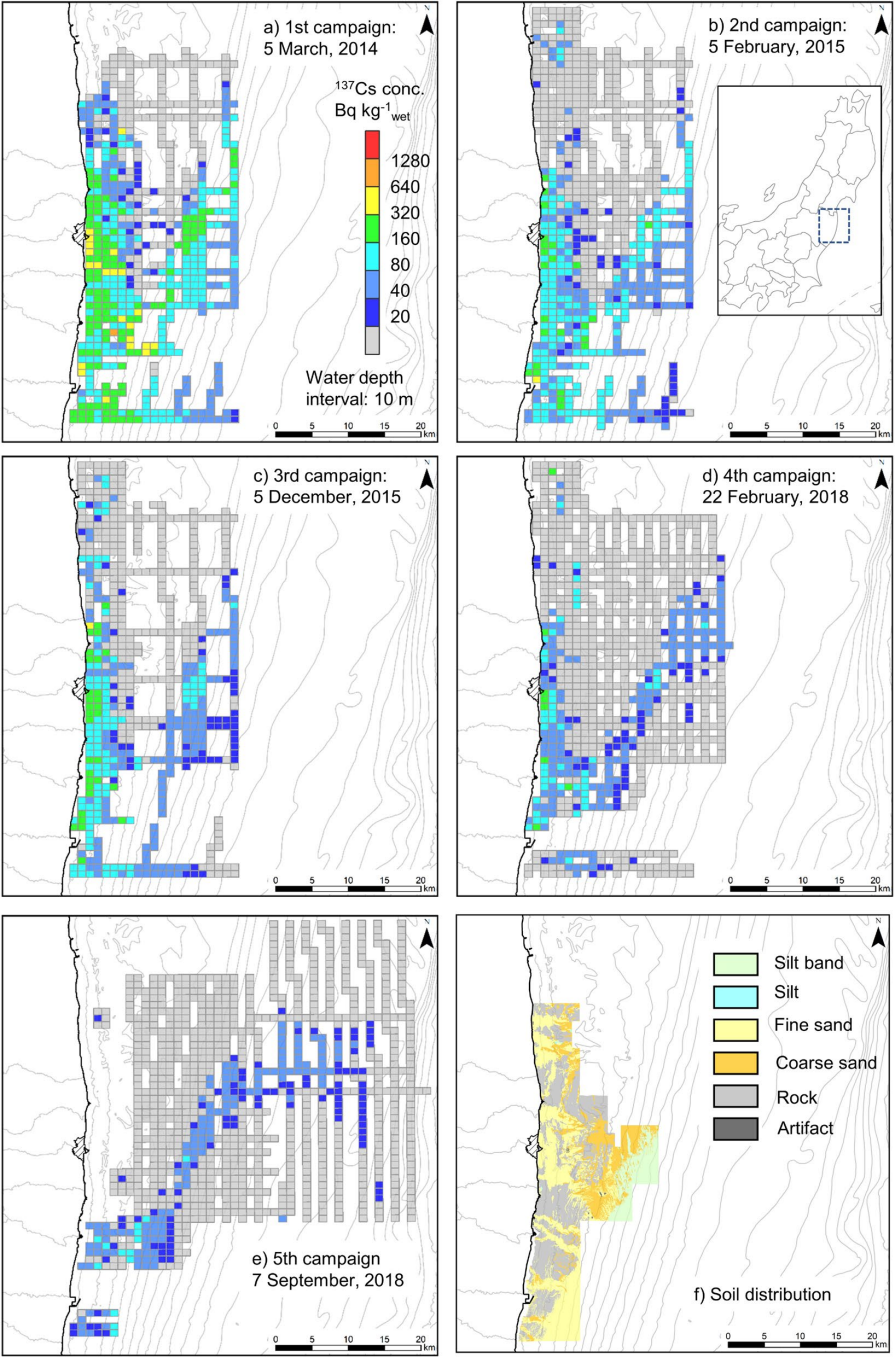
Contour maps of ^137^Cs distribution by mesh averaging method. (**a**–**e**): ^137^Cs distribution by towed radiation survey and (**f**) sediment distribution map by a bathymetric survey and sonic prospecting. Map created with ESRI ArcGIS 10.7.1. Basemap sources: M7000 Digital Bathymetric Chart (Shapefile) published by Marine Information Research Center.

Secondly, the mesh averaging map was described. The averaged detector count rate within a 1 km × 1 km mesh (approximately 100 s measurement times) was converted to ^137^Cs concentration to obtain a sufficiently low DL (approximately 10 Bq kg^−1^_wet_). The ^137^Cs contour maps of the five towed radiation survey campaigns are shown in Fig. 2. The survey area without the fifth campaign (2018) was almost the same area. The fifth campaign was conducted in an area 20 km from land. In addition, an area measured for soil distribution via bathymetric survey and sonic prospecting was part of the towed radiation survey area. The ^137^Cs concentration tended to decrease with the time elapsed since the accident, as shown in Fig. 2a–e. Throughout all the campaigns, the ^137^Cs concentration south of the FDNPP was relatively higher than that in the north. The ^137^Cs distribution at the water surface a month after the accident, as reported by Inomata et al. (2014), suggested that the contaminated water flowed toward the south from the FDNPP^16^. The distribution of ^137^Cs concentration at the seafloor was estimated to depend on the flow direction of the contaminated water immediately after the accident. The another characteristic was the area with a relatively high concentration, which lies from 20 km east of the FDNPP to 50 km northeast of the FDNPP. The soil distribution in this area revealed a thin silt layer of approximately 10 cm on the flat bedrock (Fig. 2f). This soil condition was defined as a silt band. The formulation mechanism of this silt band is related to the ocean current and the topography, as found by a simulation study conducted with a comprehensive dynamic model ten months after the accident^17^. The ^137^Cs concentration in the silt band (160–400 Bq kg^−1^_wet_) was lower than that at the estuaries (320–1000 Bq kg^−1^_wet_), as shown in Fig. 1a. By contrast, the ^137^Cs concentration in the coarse sand and rock areas was relatively low throughout all the campaigns.

### Validation of towed radiation survey

**Figure 3:**
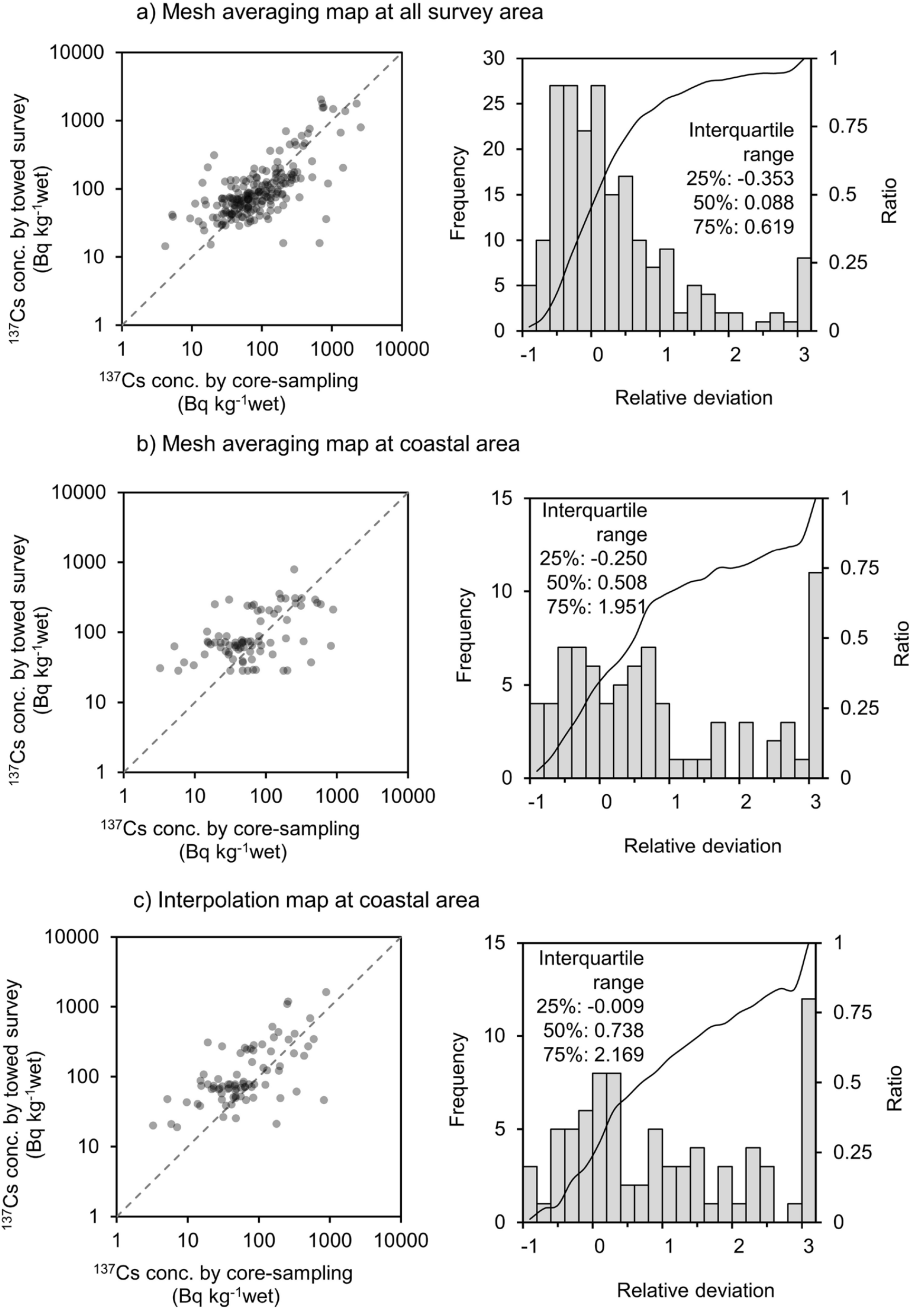
The comparison between ^137^Cs concentration of sediment core sample with towed radiation survey for validation of the towed radiation survey. (**a**) all survey area of all campaign, (**b**) limitation of the coastal area of the mesh averaging map and (**c**) limitation of the coastal area of the interpolation map.

In this section, the results of the towed radiation survey are discussed quantitatively. The ^137^Cs distribution map created via mesh averaging and the measurement results of actual sediment core samples were compared to evaluate the temporal change in the ^137^Cs concentration quantitatively. The numbers of sediment core samples are shown in Table 1. The mean ^137^Cs concentration from the surface to 10 cm depth was calculated in consideration of the detectable sediment depth of the radiation of the detector. The concentration value was corrected to the end of the end date of towed radiation survey campaign by taking into consideration of ^137^Cs half-life. A scatter diagram and a histogram of the relative deviation (*RD*) of the sediment sample and towed radiation survey results at the same locations are shown in Fig. 3a. The comparison results of each campaign are shown in Fig. S1. The plot of the scatter diagram is distributed around y = x. The peak top of the histogram of *RD* is around zero, and its 50% interquartile range (median) is 0.088. The 25% and 75% interquartile ranges are − 0.353 and 0.619, respectively.

There are several reasons for the gap between the towed radiation survey and the sediment sampling measurement. The representativeness of the sediment samples was most effective because the towed radiation survey results were evaluated by comparing them with the 1 km mesh mean and one-point soil sampling data in this study. The coincidence of towed radiation survey and sediment sample measurement was hard to determine in areas with heterogeneous ^137^Cs concentrations. The comparison result for the coastal areas, which comprise the soil distribution survey area, is shown in Fig. 3b. The comparison result varied widely. This may have been due to the heterogeneous ^137^Cs concentration in the coastal areas, as stated by past research^7,18,19^. To reinforce this situation, the comparison result between the interpolation map, which is shown in Fig. 1, and the sediment sample measurement was similar to that of the mesh averaging map, as shown in Fig. 3c. This comparison result also varied widely.

Other reason for the gap between the towed radiation survey and the sediment sampling measurement is the differences in the calibration conditions, including the ^137^Cs depth profile, water content, soil distribution, and the distance between the detector and the sediment surface. Regarding the distance between the detector and the sediment surface, Ounishi et al. (2016) concluded from a simulation study that the detector response at 10 cm burial depth is twice that at the sediment surface^14^. The unevenness of ^137^Cs distribution maps generated via towed radiation surveys is included in various factors, and this uncertainty should be considered when evaluating the ^137^Cs distribution derived from the FDNPP accident. Generally, comprehensive assessment using multiple data has a smaller influence on this uncertainty than does point-wise evaluation.

### Temporal change and soil distribution

**Figure 4:**
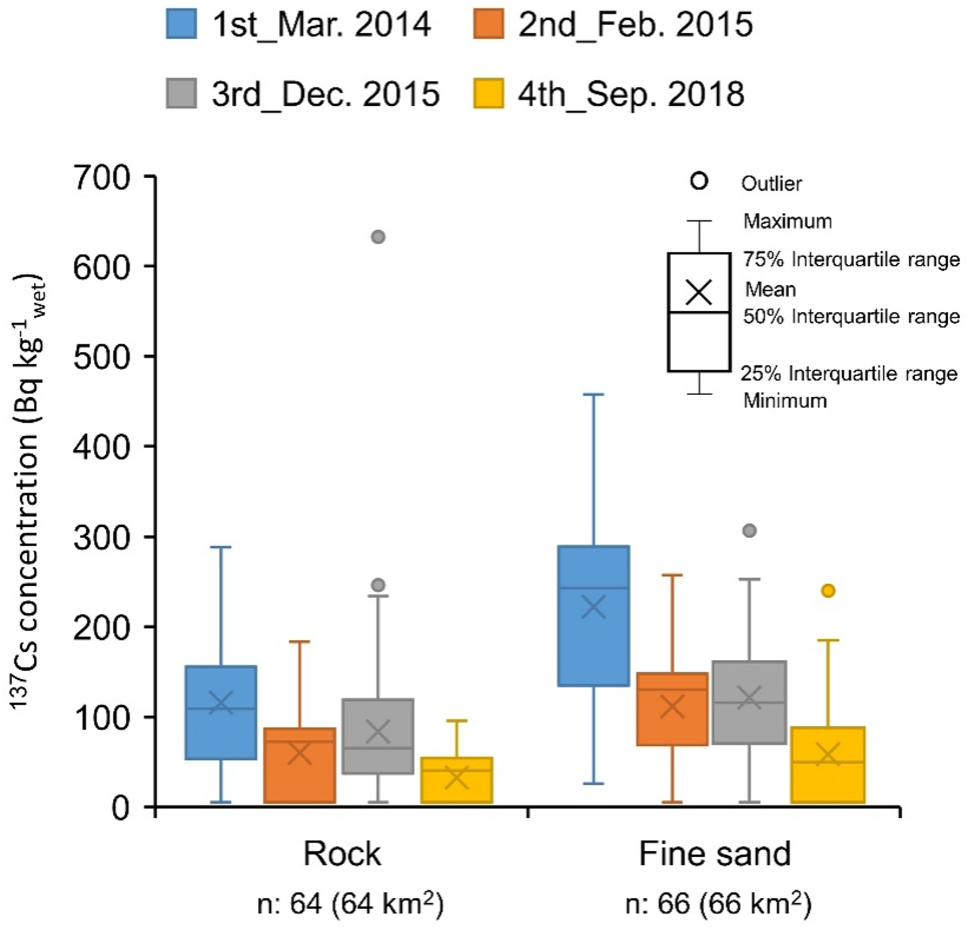
Temporal change of ^137^Cs concentration at different geological (rock and fine sand).

Temporal changes of ^137^Cs concentration should be examined to predict future radionuclides concentration and help identify the mechanism of the environmental behavior of general pollution materials. Figure 4 shows box plots of the ^137^Cs concentration in the rock and fine sand areas, which are based on the dataset of the mesh averaging map (Fig. 2). For this analysis, the mesh of the measured data from the first campaign to the fourth was extracted (rock area: n = 64, fine sand area: n = 66). The ^137^Cs concentration tended to decrease with time despite the soil distribution. The increase in the third campaign reflected a large increment in the ^137^Cs inflow from the terrestrial area through Ukedo River caused by a remarkable typhoon in September 2015 (Etau)^20^. The ^137^Cs concentration in the rock area was half of that in the fine sand area throughout all the campaigns. Here a “rock area” is defined as an area where rock occupied more than 80% of the soil distribution within a 1 km × 1 km mesh (the unit of the towed radiation survey map). The less than 20% soil distribution without rock was the overestimation factor of ^137^Cs concentration, as the boundary between rock and sand was surmised to accumulate the fine sand. For example, a plot of more than 600 Bq kg^−1^_wet_ in the third campaign (Fig. 4) was identified in the Tomioka River area, as shown in Fig. 1c. Finally, the ^137^Cs concentration in the rock area was smaller than that in the fine sand area.

## Summary and conclusion

In this study, the ^137^Cs concentration in the surface sediment off the shore of Fukushima was visualized on the basis of towed radiation survey data. The characteristics of the formation of ^137^Cs anomaly at estuaries were discussed using a contour map of ^137^Cs concentration combined with water depth. The created map was confirmed to be comparable with the actual sediment sample. The map generated using the towed radiation survey represented the ^137^Cs concentration distribution as the position resolution of a 1 km mesh. However, ^137^Cs distribution heterogeneity and high ^137^Cs concentration in deeper layers (> 10 cm from sediment surface) at estuaries led to the under- or over-estimation of the actual ^137^Cs concentration. Furthermore, the ^137^Cs concentration was found to decrease with elapsed time in consideration of such uncertainty, and the ^137^Cs concentration in fine sand areas was larger than that in rock areas. This visualized map is expected to help reveal the mechanism of the environmental behavior of radionuclides in evaluation of the additional exposure dose after the FDNPP accident. These data will be published in the Japan Atomic Energy Agency's database for the study of environmental radiation and exposure dose^21^.

## Material and methods

### Towed radiation survey system

**Figure 5:**
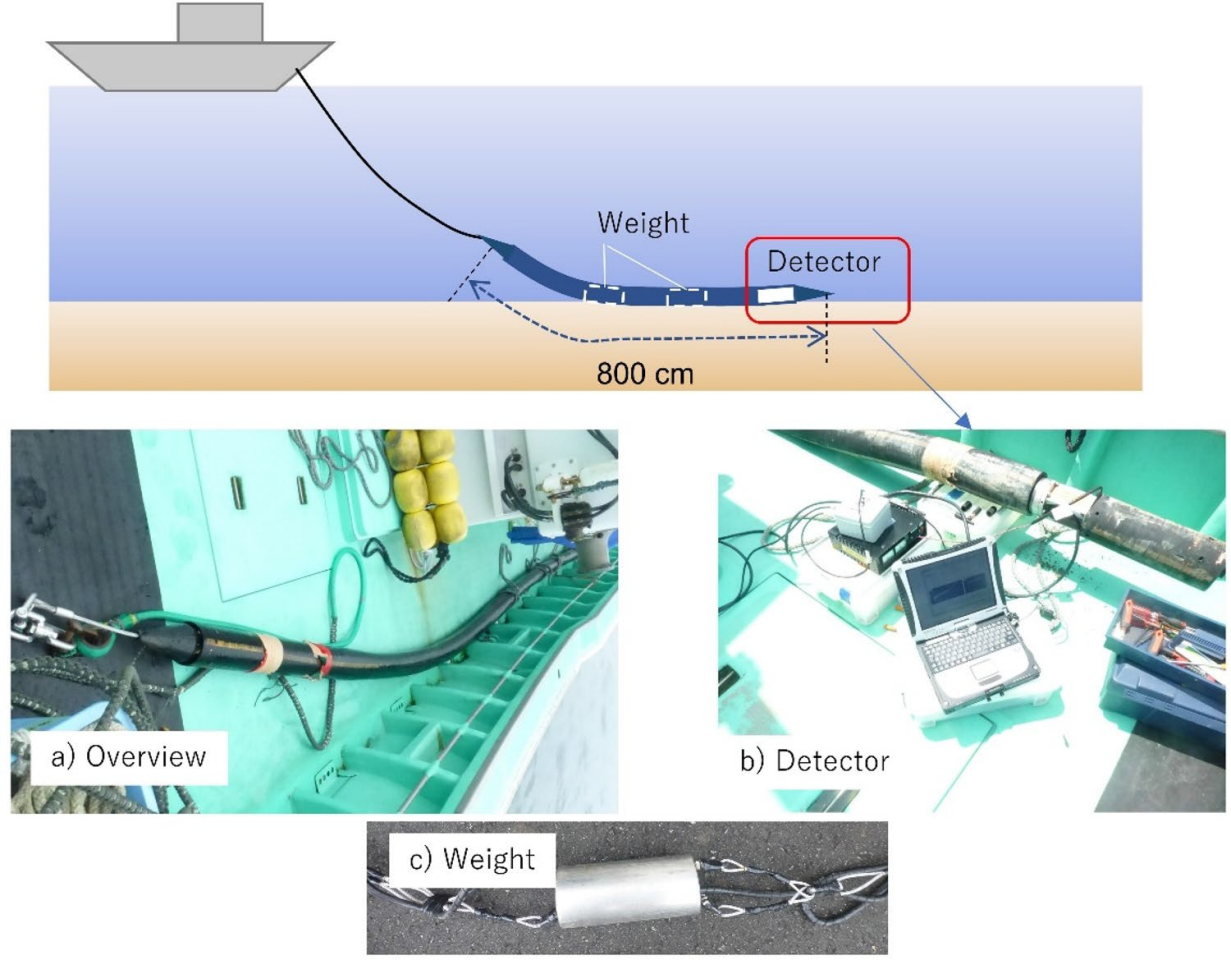
Image of the towed gamma-ray detection system.

Two NaI (Tl) scintillation detectors were used for the towed radiation survey system: a small version (dimensions: 3″$\upphi$ × 3″H) and a large type (dimensions: 3″$\upphi$ × 6″H). Figure 5 shows an overview and a photo of the towed radiation survey system. This system is 8 m long and has an external diameter of 0.145 m. The towed radiation survey system has enough underwater weight (115 kg) to maintain contact with the seafloor at an operational speed of 2 knots^14^. The spectrometer was calibrated to measure the gamma-ray spectrum between 0.8 and 1.8 MeV over 1024 channels and has a resolution of 7.0% at 0.662 MeV. The radiation detector is covered with a rubber hose to reduce its risk of snagging and protect it from abrasion and impact damage as it follows the undulations of the seafloor.

### Data acquisition

**Figure 6:**
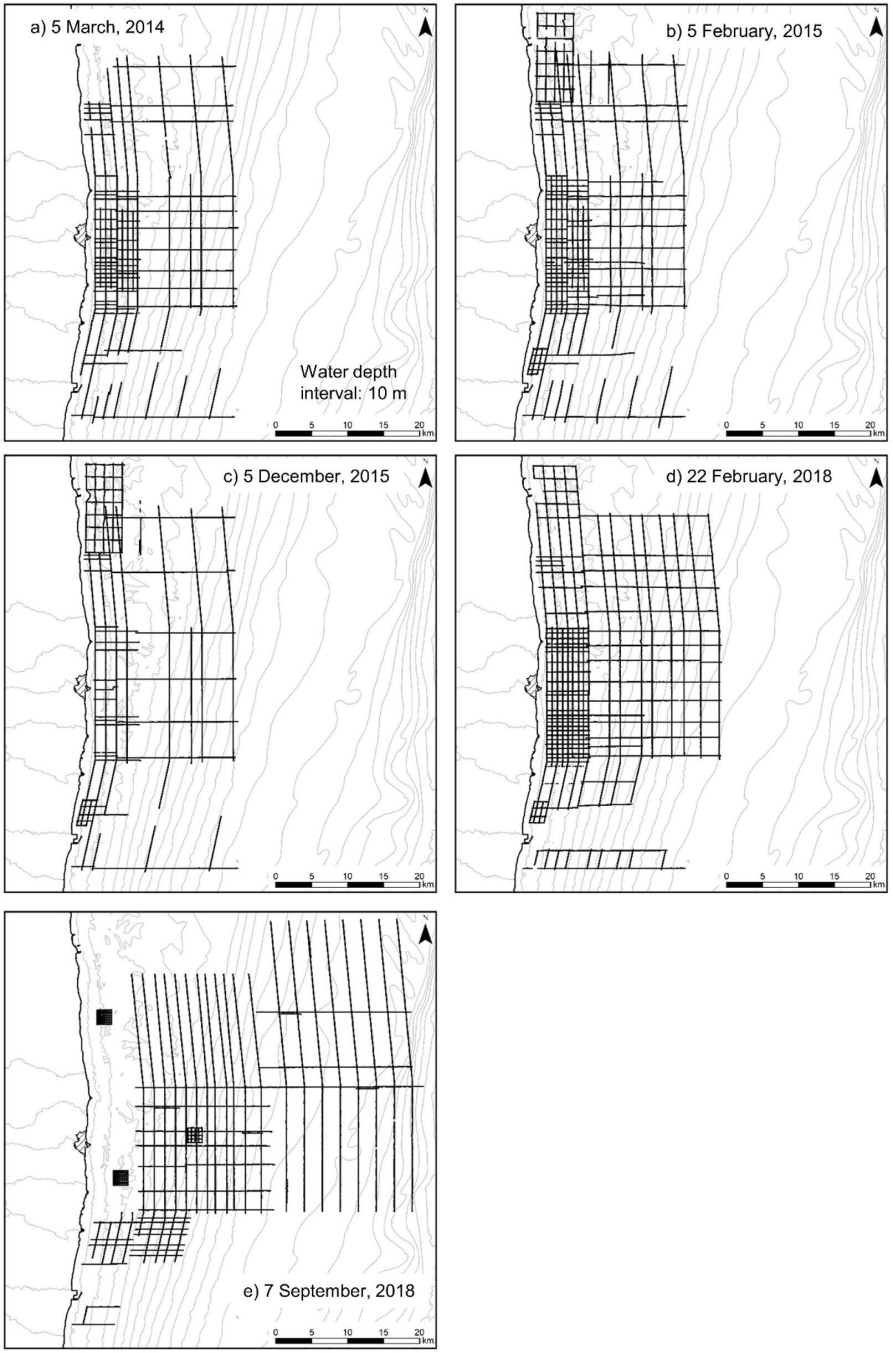
The track line of the towed radiation survey. Map created with ESRI ArcGIS 10.7.1. Basemap sources: M7000 Digital Bathymetric Chart (Shapefile) published by Marine Information Research Center.

The gamma-ray spectrum was acquired every second, and the temperature and depth were recorded by a depth sensor at the same time. The readings were synchronized with time via the global positioning system (GPS) receiver installed on the ship. The spectrum and the GPS data (date, time, latitude, longitude, and height above ellipsoid) were recorded every second. The actual detector position was estimated from the water depth, length of supplied wire, and GPS position data from the ship. The survey lines for each campaign are shown in Fig. 6. The intervals between the survey lines were set as approximately 1–2.5 km in consideration of the location of the river mouth and the seafloor characteristics, such as silt and sand. These monitoring data were obtained by the NRA and the National Maritime Research Institute as part of the national radiation survey project^22^.

For data cleansing, two criteria were set on the basis of the detector count rate. One criterion was less than the background count rate, which was obtained at 10 m locations from the sea surface at 20 m water depth. The reason of these data was that the detector was away from the seafloor. The other criterion was the lack of a radiocesium region-of-interest (ROI) peak in the obtained gamma spectrum caused by a high count rate. At the time of data acquisition, these data were deemed to indicate the pile-up loss of signal treatment or effects of detector noise. These data were excluded from the analysis.

### Gamma spectrum analysis

**Figure 7:**
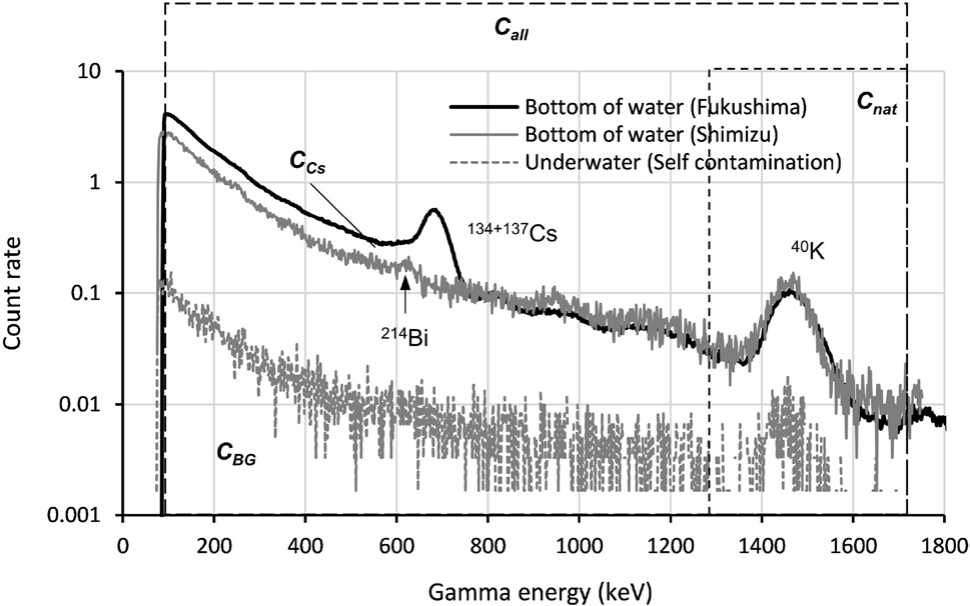
Gamma spectrum of the towed radiation survey system and the setting of the region of interest. The measurement time of this spectrum was 600–1200 s.

In this study, the gamma spectrum at the seafloor was analyzed according to the method suggested in an International Atomic Energy Agency report^23^. A typical gamma spectrum collected at the seafloor is shown in Fig. 7. The counting peak of radiocesium was found in this gamma spectrum. The following sources of background radiation influence radiocesium counting: (1) the natural nuclides in the detector crystal and the structure of the detector system and (2) the natural nuclides in the bottom sediment.

Regarding the first disturbance factor, the background counting rate (*C*_*BG*_) was obtained at 10 m locations from the sea surface at 20 m water depth. An example of the background spectrum is shown in Fig. 7. For the second disturbance factor, the gamma spectrum was obtained from an area with low-level radiocesium contamination 300 km from the FDNPP. The maximum ^137^Cs concentration in the sediment at this location was 0.34 Bq kg^−1^. This value was negligible compared with the approximately 100–300 Bq kg^−1^ of ^40^ K concentration in this region. Spectrum data of 600 s measurement time (which was set to reduce counting errors) were obtained at ten points around this area. Detailed information about this natural background data is shown in Fig. S2. As indicated in Fig. 7, we set more than 1300 keV as the ROI for the natural background. With use of this natural background spectrum (which is deducted the *C*_*BG*_ from the natural background spectrum), the index of the natural background (*I*_*BG*_) was set to the ratio of the total counting rate ($C_{all}^{\prime }$), and more than 1300 keV was set as the ROI for the natural background ($C_{nat}^{\prime }$) of this natural background spectrum (*I*_*BG*_ = $C_{all}^{\prime }$/$C_{nat}^{\prime }$). The counting rate of radiocesium was calculated as

1 $$ C_{Cs} = C_{all} - C_{BG} - C_{nat} \cdot I_{BG} . $$

This following typical equation is applicable to calculation of the DL:

2 $$ DL = \frac{k}{2}\left\{ {\frac{k}{{t_{s} }} + \sqrt {\left( {\frac{k}{{t_{s} }}} \right)^{2} + 4n_{b} \left( {\frac{1}{{t_{s} }} + \frac{1}{{t_{b} }}} \right)} } \right\}, $$

where *n*_*b*_ is defined as (*C*_*BG*_ + *C*_*nat*_* I*_*BG*_), *k* is the standard deviation of the Gauss distribution (*k* = 3), *t*_*s*_ is the measurement time, and *t*_*b*_ is the background measurement time.

### Sediment sample

**Figure 8:**
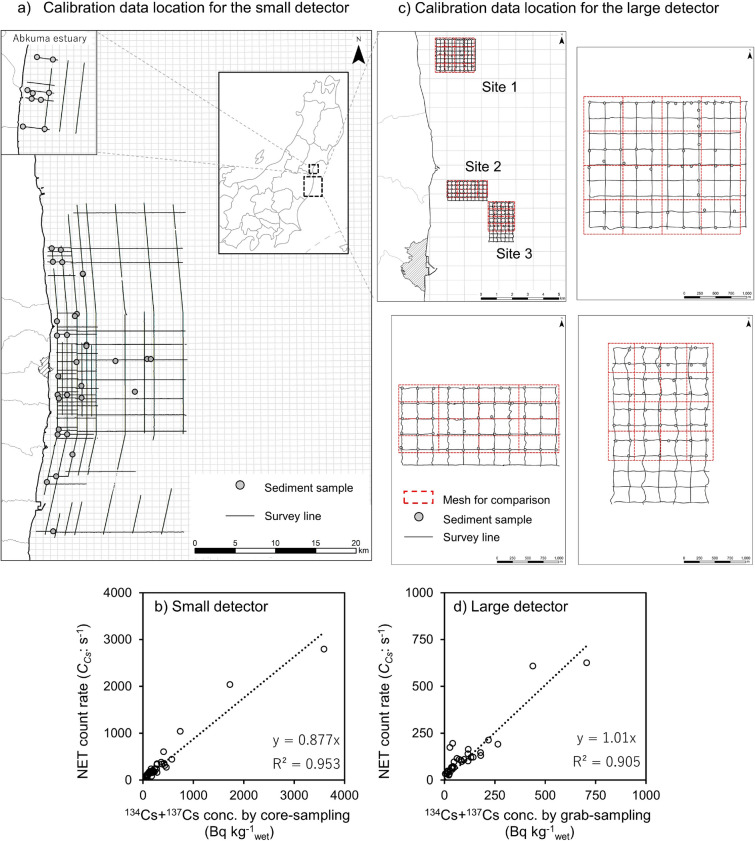
The survey line of the towed radiation survey system and the locations of (**a**) sediment core and (**b**) grab samples for obtaining the calibration data. The linearity of radiocesium concentration using (**c**) small detector and (**d**) large detector. Map created with ESRI ArcGIS 10.8.0. B Basemap sources: ESRI ArC GIS on-line ((**c**) Esri Japan).

For calibration of the small detector used in the towed radiation survey, 39 sediment core samples (length 10–40 cm) were collected from the area of the towed radiation survey in 2014, as shown in Fig. 8. The sampling location was selected among the survey lines for the towed radiation survey. The samples were collected with an HR-type core sampler (Rigo Co. Ltd., Saitama, Japan), sectioned into 5 cm layers, dried, and measured for radiocesium activity concentration (on the wet basis: Bq kg^−1^_wet_) via gamma spectrometry using a p-type high-purity germanium detector (Seiko EG&G Co. Ltd., Tokyo, Japan) that was calibrated by standard gamma sources. For calibration of the large detector used in the towed radiation survey, 150 sediment grab samples (weight 0.5–1 kg) were collected from the area of the towed radiation survey in 2020, as shown in Fig. 8b and Table 1. The sampling location was selected around an intersection of the survey lines for the towed radiation survey. The samples were collected with an Ekman-Berge bottom sampler (Rigo Co. Ltd., Saitama, Japan), portioned, dried, and measured for radiocesium activity concentration (on the wet basis: Bq kg^−1^_wet_) via gamma spectrometry using a p-type high-purity germanium detector. For validation of the towed radiation survey, 21–59 sediment core samples (length 30–40 cm) were collected from the area of the towed radiation survey between 2014 and 2018 (Table 1). These samples were treated and measured in the same manner as were the calibration samples.

### Calibration

For conversion from counting rate to ^137^Cs concentration, two types of towed radiation survey systems were calibrated using field data obtained off the coast of Fukushima. In 2014, a towed radiation survey with a small detector was conducted within the survey lines, as shown in Fig. 8a. The radiocesium measurement result from the sediment surface (0 cm) to 10 cm of the sediment core sample, which was collected from the survey lines, was averaged for comparison with *C*_*Cs*_, which was averaged in 100 m at the center of the sampling location. A scatter diagram of the radiocesium measurement result from the sediment surface to 10 cm of the sediment core sample and *C*_*Cs*_ is shown in Fig. 8b. In this figure, the actual sample measurement and *C*_*Cs*_ correlate well from 30 Bq kg^−1^ to 3500 Bq kg^−1^. This approximate straight line inclination was defined as a conversion factor (*CF*: 0.877 cps Bq^−1^ kg_wet_). In 2020, a towed radiation survey with a large detector was conducted within the survey lines, as shown in Fig. 8c. The radiocesium measurement result from the sediment surface (0 cm) to 10 cm of the grab sample collected at an intersection of the survey lines was prepared for comparison with *C*_*Cs*_. We selected a grab sample instead of a core sample because many samples are needed due to the low radiocesium concentration in the sediment. The *C*_*Cs*_ and radiocesium concentration of the actual sample in the selected grid (marked by red line in Fig. 8c) were averaged and compared. A scatter diagram of the radiocesium measurement result in the sediment surface of the grab sample and *C*_*Cs*_ is shown in Fig. 8d. In this figure, the actual sample measurement and *C*_*Cs*_ correlate well from 30 Bq kg^−1^ to 750 Bq kg^−1^. This approximate straight line inclination was defined as a conversion factor (*CF*: 1.01 cps Bq^−1^ kg_wet_).

In addition, the ^40^ K calibration of the detector was conducted by substituting *C*_*nat*_ for *C*_*Cs*_ in the above calibration methods. A scatter diagram of the *C*_*nat*_ of the large detector towed radiation survey is shown in Fig. S3. In this comparison, the ^40^ K measurement data of the grab sample and averaged *C*_*nat*_ (red line in Fig. 8c) were compared. The ^40^ K distribution map was created using this parameter, as shown in Fig. S3.

The concentration of ^137^Cs was calculated and corrected with the physical half-life of radiocesium (^134^Cs: 2.0652 years, ^137^Cs: 30.167 years) and the ratio of both cesium (^134^Cs/^137^Cs) on the accident date (March 15, 2011). The detector energy responses of ^134^Cs and ^137^Cs were assumed to be equal. With use of these parameters and Eq. (2), the DLs of the small and large detectors were determined to be 8.9 and 5.0 Bq kg^−1^, respectively. In this calculation, the measurement time is evaluated at 100 s.

The radiocesium in the sediment more than 10 km off the coast of Fukushima is spread approximately 10 cm from the sediment surface, as indicated by considerable field data^6,24, 25^. The radiocesium depth profile in nearshore and estuary areas is complex; a radiocesium peak has been observed at a depth of over 50 cm^7^. Towed radiation survey cannot evaluate all types of radiocesium depth profiles. In this study, the averaged radiocesium concentration from the sediment surface to 10 cm depth was the target of assessment.

### Mesh averaging and interpolation

Mapping was performed by supplementing unmeasured areas via two methods. The first was a simple technique for the mean in a 1 km × 1 km mesh. The gamma spectrum data in the mesh were summed, and the counting rate of each ROI (*C*_*nat*_, *C*_*Cs*_, and *C*_*all*_) was calculated. Afterward, these ROIs were converted to ^137^Cs concentrations as averaged data in the mesh. This map was created using the software ArcGIS 10.7.1.

The second method was interpolation of the survey results. Various methods have been proposed for interpolation, such as kriging and spline approaches. In this study, the inverse distance weighting (IDW) method, wherein weights are assigned to the values of measurement points linearly and in inverse proportion to distance, was applied to the survey data. The IDW method is easy to use in analyzing large amounts of data because of the simplicity of its parameter setting^23^. This interpolation processing was conducted using ArcGIS. The spatial resolution of the resulting contour map was 250 m × 250 m, and the maximum distance of the search radius was 360 m.

### A bathymetric survey and sediment distribution map

Tsuruta et al. (2017) created a geological map of the Ukedo River estuary, which is located 7 km north of the FDNPP^7^. In the national project covered by the present study, a bathymetric survey of approximately 500 km^2^ at Fukushima off-shore was conducted using the technique of Tsuruta et al. (2017)^7^. A bathymetric survey using sonic prospecting was performed to visualize the characteristics of the seabed sediment from 2013 to 2020. PDR-1300(W) (SenbonDenki Numazu, Shizuoka, Japan; depth range of up to 2 m) and Sonic 2024 (R2SONIC, Texas, USA; sectors deeper than 2 m) echo sounders were selected for the bathymetric survey. A bathymetric map with a resolution of 2–3 m was produced using the commercial software Marine Discovery (Ocean High Technology Institute Inc., Tokyo, Japan). Sonic prospecting was conducted using a System 3000 (L-3 Klein, New Hampshire, USA; depth range of up to 3 m) and a 2000-DSS (EdgeTech, Massachusetts, USA; sectors deeper than 3 m), and it involved a full-coverage side-scan sonar survey and a sub-bottom profile survey. The sonic prospecting data were processed using the software CleanSweep (Oceanic Imaging Consultants, Hawaii, USA). Side-scan sonar mosaics with a 0.5 m resolution were obtained. The backscatter intensity value of these mosaics can be used to semi-qualitatively identify the seafloor type (e.g., rock or sediment). Seabed sediment sampling was performed at 18 locations by using a Smith-McIntyre grab sampler (Rigo Co. Ltd., Saitama, Japan), and the sediment types, namely, silt (including clay), sand (fine, medium, and coarse), and granule, were confirmed via visual observation. The backscatter intensity values were compared with the results of seabed sediment sampling and the geological map, and the seafloor types in the investigation area were predicted. The areas with high backscatter intensities correspond to bedrock. The remaining areas, which were considered to be the distribution areas of the seabed sediment, were qualitatively classified into three grain-size regions on the basis of the backscatter intensity features: coarse sand–granule, fine–medium sand, and silt. The major factors used for this classification are as follows: coarse sand–granule (high and spotted backscatter intensity), fine–medium sand (medium–low and pervasive homogeneous backscatter intensity), and silt (including clay; very low and pervasive backscatter intensity).

The sub-bottom profile data were collected using a Bathy-2010 (SyQwest, Osaka, Japan) up to 3 m depth, and using an EdgeTech 2000-DSS in sectors deeper than 3 m. These data were processed using the Marine Discovery 8 software package (Ocean High Technology Institute Inc.). The sub-bottom profilers were roughly divided into bedrock and sediment areas according to the intensity of reflected waves. The thickness of the seabed sediment overlying the bedrock was estimated on the basis of an interpretation of the boundary between the seabed sediment and bedrock.

## Supplementary Information

Supplementary Information.

## References

[1] Katata G (2014). Detailed source term estimation of the atmospheric release for the Fukushima Dai-ichi Nuclear Power Station accident by coupling simulations of atmospheric dispersion model with improved deposition scheme and oceanic dispersion model. Atomos. Chem. Phys..

[2] Saito K (2019). Summary of temporal changes in air dose rates and radionuclide deposition densities in the 80 km zone over five years after the Fukushima Nuclear Power Plant accident. J. Environ. Radioact..

[3] Sanada Y, Urabe Y, Sasaki M, Ochi K, Torii T (2018). Evaluation of ecological half-life of dose rate based on airborne radiation monitoring following the Fukushima Dai-ichi nuclear power plant accident. J. Environ. Radioact..

[4] Nuclear Regulation Authority, *Monitoring information of environmental radioactivity level, readings of sea area monitoring*. https://radioactivity.nsr.go.jp/en/list/205/list-1.html. Accessed on 5 July 2021.

[5] Buesseler K (2017). Fukushima Daiichi–derived radionuclides in the ocean: Transport, fate, and impacts. Ann. Rev. Mar. Sci..

[6] Otosaka S, Kato Y (2014). Radiocesium derived from the Fukushima Daiichi Nuclear Power Plant accident in seabed sediments: Initial deposition and inventories. Environ. Sci. Process. Impacts.

[7] Tsuruta T, Harada H, Misonou T, Matsuoka T, Hodotsuka Y (2017). Horizontal and vertical distributions of ^137^Cs in seabed sediments around the river mouth near Fukushima Daiichi Nuclear Power Plant. J. Oceanogr..

[8] Otosaka S (2017). Processes affecting long-term changes in ^137^Cs concentration in surface sediments off Fukushima. J. Oceanogr..

[9] Takata H (2020). Suspended particle-water interactions increase dissolved ^137^Cs activities in the nearshore seawater during Typhoon Hagibis. Environ. Sci. Technol..

[10] Jones DG (2001). Development and application of marine gamma-ray measurements: A review. J. Environ. Radioact..

[11] Jones DG, Roberts PD, Miller JM (1988). The distribution of gamma-emitting radionuclides in surface subtidal sediments near the Sellafield Plant. Estuar. Coast. Shelf Sci..

[12] Kumagai H, Iwase R, Kinoshita M, Machiyama H, Hattori M, Okano M, Adrovic F (2012). Environmental Gamma-Ray Observation in Deep Sea. Gamma Radiation.

[13] Thornton B (2013). Distribution of local ^137^Cs anomalies on the seafloor near the Fukushima Dai-ichi Nuclear Power Plant. Mar. Pollut. Bull..

[14] Ounishi S, Thornton B, Kamada S, Hirao Y, Ura T, Odano N (2016). Conversionfactoranduncertaintyestimationforquantification of towed gamma-ray detector measurements in Tohoku coastal waters. Nucl. Instrum. Methods Phys. Res. A.

[15] Tanaka K, Iwatani H, Sakaguchi A, Fan Q, Takahashi Y (2015). Size-dependent distribution of radiocesium in riverbed sediments and its relevance to the migration of radiocesium in river systems after the Fukushima Daiichi Nuclear Power Plant accident. J. Environ. Radioact..

[16] Inomata Y (2014). Distribution of radionuclides in surface seawater obtained by an aerial radiological survey. J. Nucl. Sci. Technol..

[17] Higashi H, Morino Y, Furuichi N, Ohara T (2015). Ocean dynamic processes causing spatially heterogeneous distribution of sedimentary caesium-137 massively released from the Fukushima Daiichi Nuclear Power Plant. Biogeosciences.

[18] Misonou, T., Tsuruta, T., Nakanishi T., & Sanada, Y., Summary of radioactive Cs dynamics studies in coastal areas and assessment of river impacts. in *Progress in Environmental Emergency Research after the Great East Japan Earthquake and Fukushima Nuclear Disaster* (ed Ohara, T.), Vol. 24, No. 2, 137–144 (Global Envirionemntal Research, 2021). http://www.airies.or.jp/ebook/Global_Environmental_Research_Vol.24No.2.pdf. Accessed at 2 July 2021.

[19] Sanada Y, Kenji M, Kotaro O, Matsuzaki K, Ogawa T, Senga Y (2018). After the FDNPS accident, this USV has used for survey of radiocesium concentration in surface waterbed. J. Adv. Mar. Sci. Technol..

[20] Nakanishi T, Sato S, Matsumoto T (2019). Temporal changes in radiocesium deposition on the Fukushima floodplain. Radiat. Prot. Dosimetry.

[21] Japan Atomic Energy Agency, 2020. *Database for radioactive substance monitoring data*. https://emdb.jaea.go.jp/emdb/en/. Accessed 1 July 2021.

[22] National Maritime Research Institute, *Report on survey on the radioactive substance in the coastal areas near Fukushima Prefecture in FY2018*. https://radioactivity.nsr.go.jp/ja/contents/15000/14752/view.html. Accessed 1 July 2021. (**in Japanese**).

[23] International Atomic Energy Agency IAEA, *Guidelines for adioelement Mapping Using Gamma Ray Spectrometry Data*. IAEA-tecdoc-1363. IAEA, Vienna (2003).

[24] Ambe D (2014). Five-minute resolved spatial distribution of radiocesium in sea sediment derived from the Fukushima Dai-ichi Nuclear Power Plant. J. Environ. Radioact..

[25] Misonou, T. *et al*., *Survey on the radioactive substance in the coastal areas near Fukushima Prefecture in FY2019 (Contract research)*. JAEA-Research 2020-008, p. 166 (2020). 10.11484/jaea-research-2020-008. (**in Japanese**).

