# Supplementary material for: Visualization of radiocesium distribution in surface layer of seafloor around Fukushima Daiichi Nuclear Power Plant
Source: Sci Rep. 2021 Nov 30;11:23175. doi: 10.1038/s41598-021-02646-9 (PMC8633336; doi:10.1038/s41598-021-02646-9)
Supplement: Supplementary file 1 — Supplementary Information. [file 41598_2021_2646_MOESM1_ESM.docx]

Supplementary material

Visualization of radiocesium distribution in surface layer of seafloor around Fukushima Daiichi Nuclear Power Plant

Yukihisa Sanada ^1*^, Yoshimi Urabe ^2^, Toshiharu misonou ^1^, Takehiko Shiribiki ^3^,

Takahiro Nakanishi^1^, Yusuke Watanabe^1^, Tadahiko Tsuruta^1^

1 Sector of Fukushima Research and Development, Japan Atomic Energy Agency, 45−169 Sukakeba, Minamisoma, Fukushima 975−0036, Japan.

2 NESI Inc, 38 Shinko-cho, Hitachinaka, Ibaraki, 312-0005, Japan

3 Sanyo Techno Marine, 1-3-17 Horidomecho, Nihonbashi, Chuouku, Tokyo, 103-0012, Japan

Corresponding author

* Yukihisa Sanada

Phone: +81−244−25−2072

e−mail address: sanada.yukihisa@jaea.go.jp


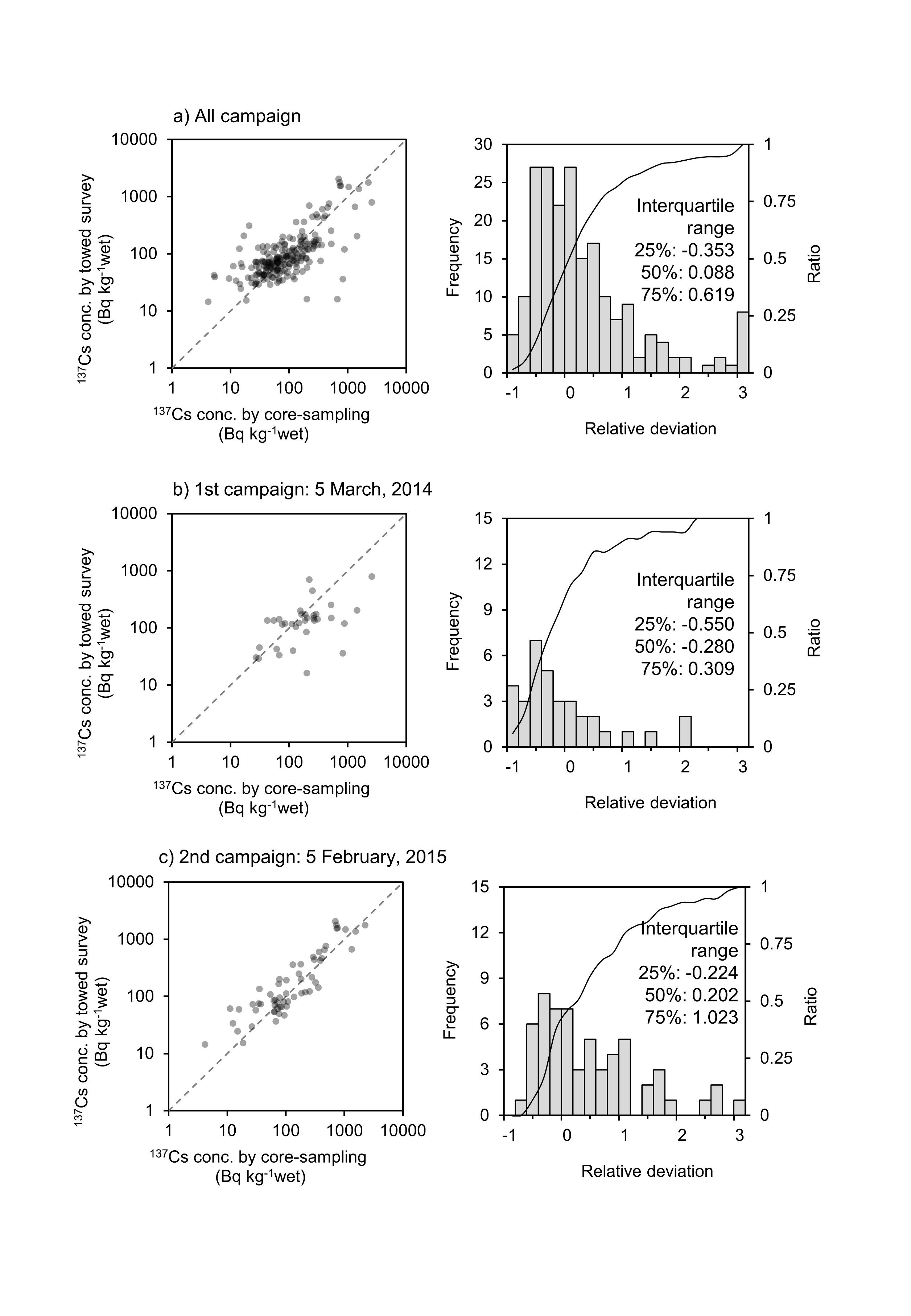
Figure S1-1. Comparison results of the towed radiation survey and core-sampling for conducting the validation of towed radiation survey. a) all campaign data, b) 1st campaign data and c) 2nd campaign data.


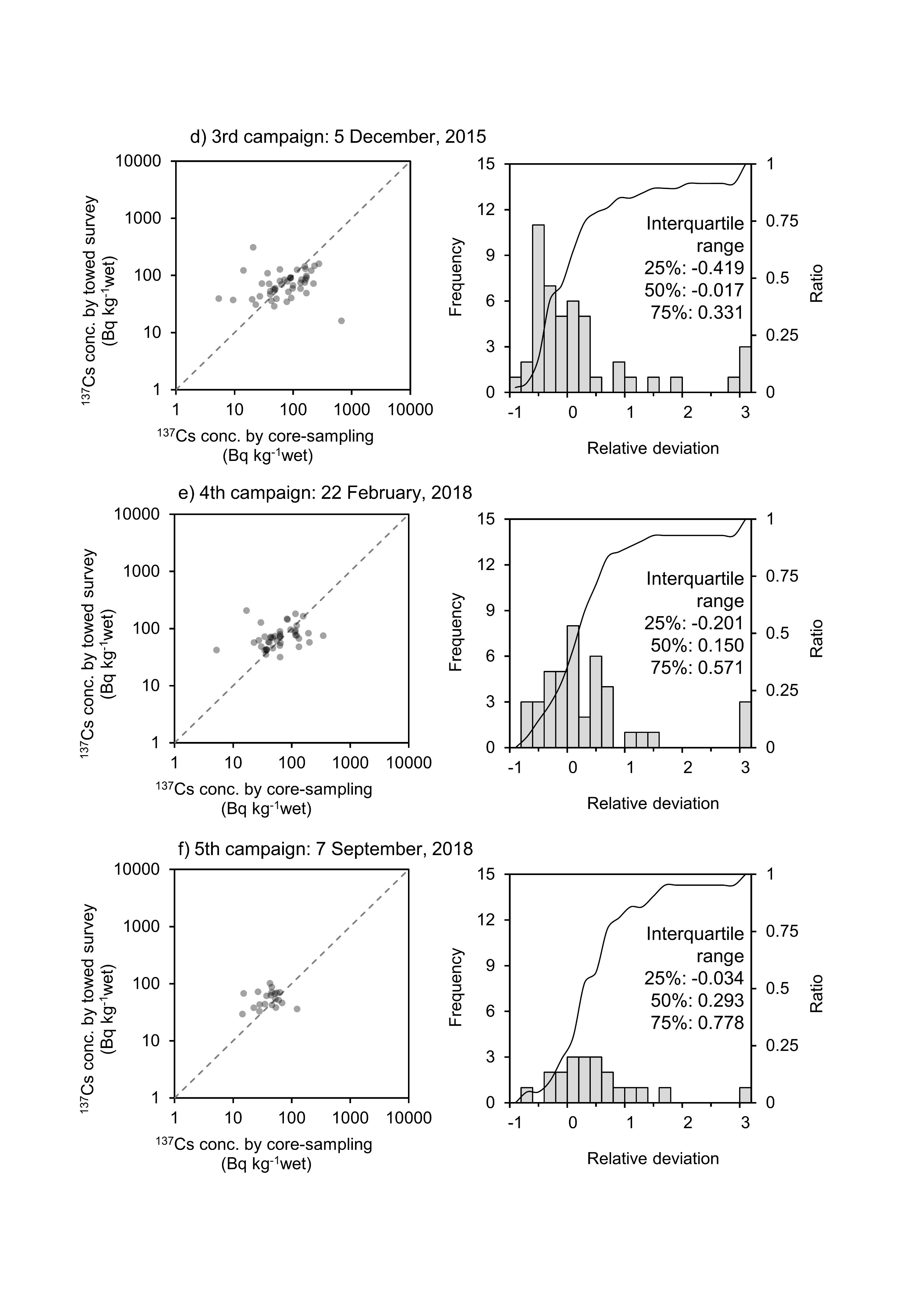
Figure S1-2. Comparison results of the towed radiation survey and core-sampling for conducting the validation of towed radiation survey. d) 3rd campaign data, e) 4th campaign data and f) 5th campaign data.


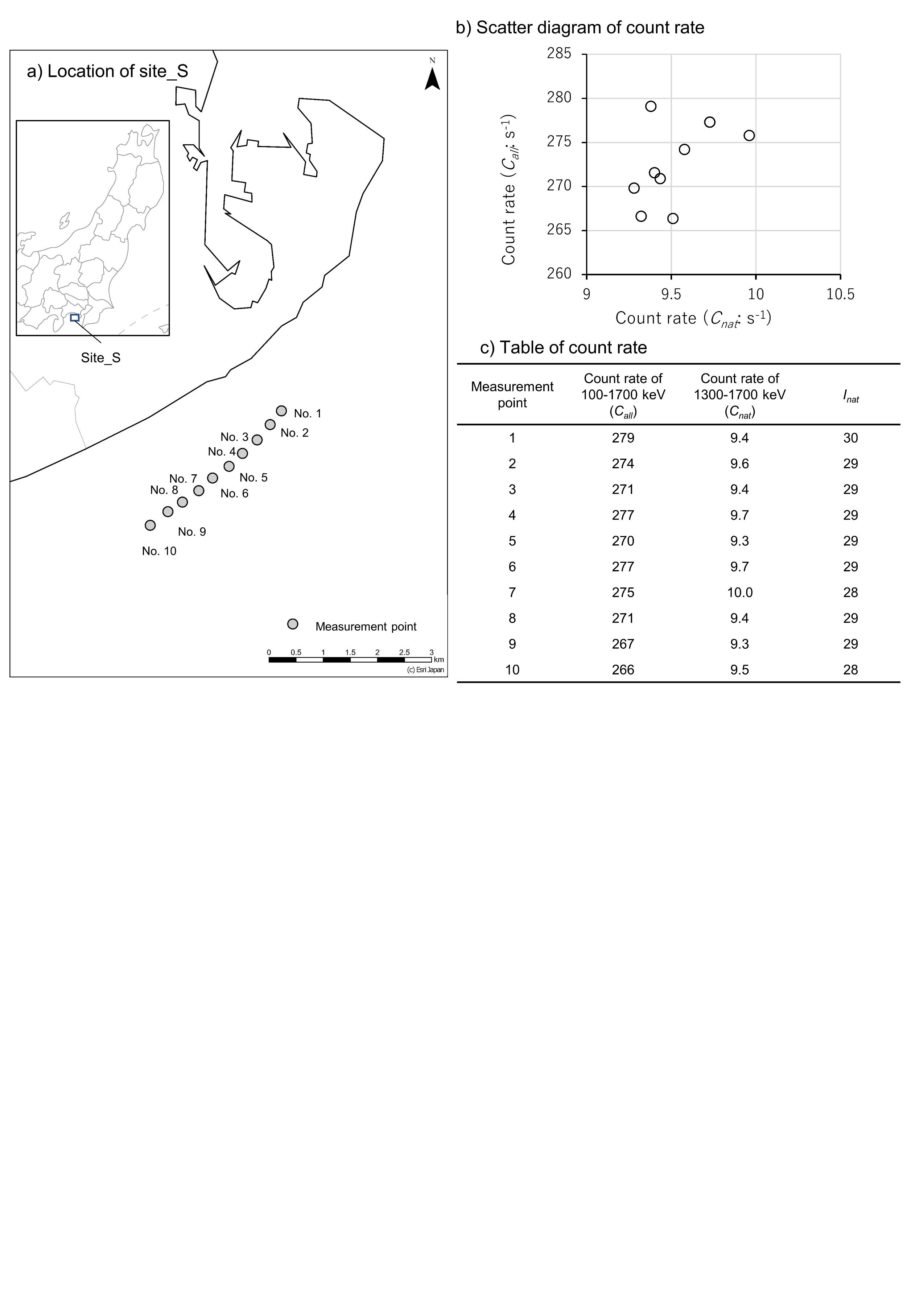
Figure. S2. Natural radionuclide for calibration of the towed radiation survey. a) Sampling location, b) scatter diagram of count rate between1300-1700 and 100-1700 keV, and c) value of *I_BG_*. Map created with ESRI ArcGIS 10.7.1. Basemap sources: ESRI ArC GIS on-line ((c) Esri Japan). .


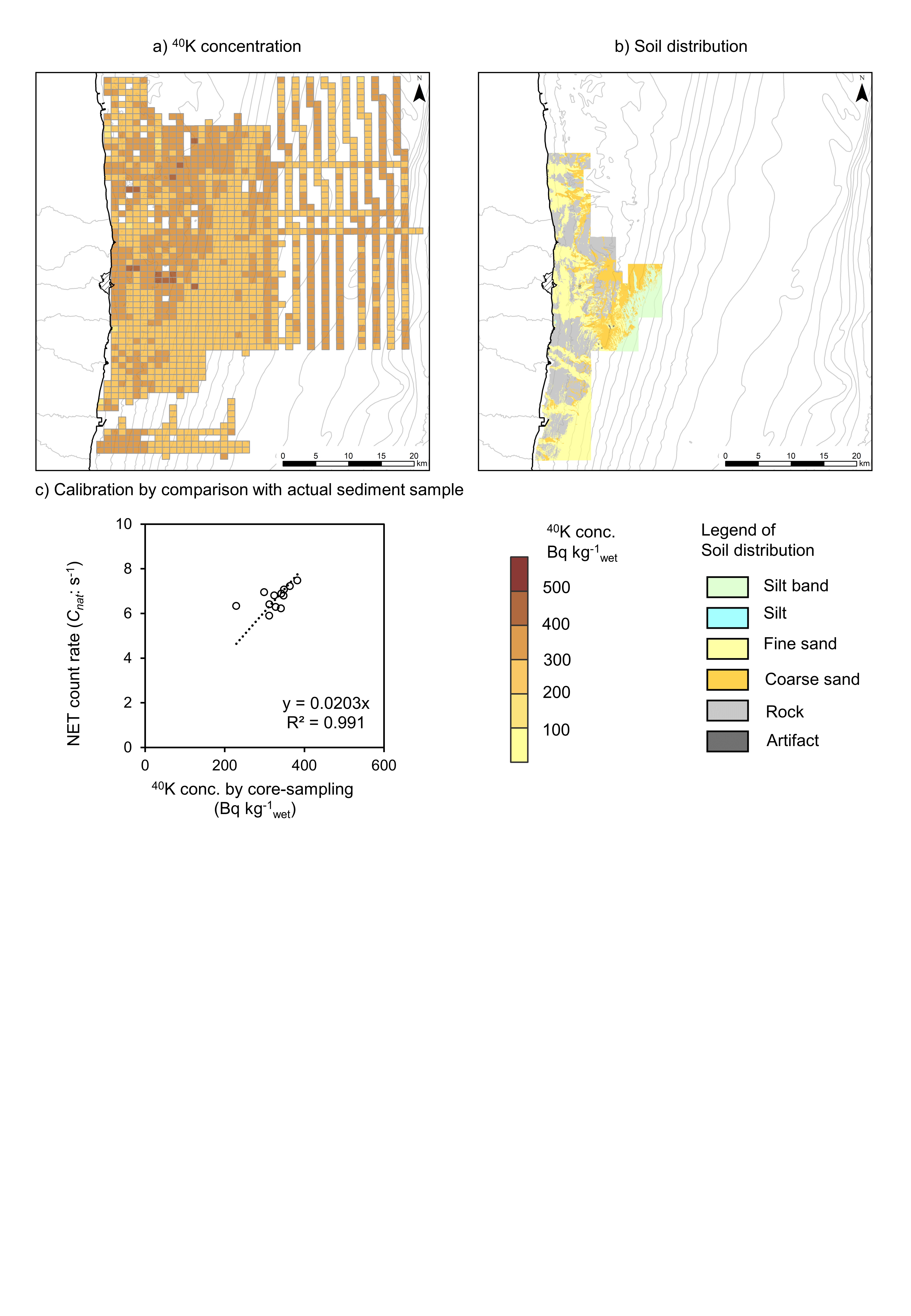


Figure. S3. ^40^K concentration map using the towed radiation survey. a) ^40^K distribution map (1 km mesh), b) geological map, and c) scatter diagram of count rate and count rate of 1300-1700 keV (*C_nat_*) for calibration. Map created with ESRI ArcGIS 10.7.1. Basemap sources: M7000 Digital Bathymetric Chart (Shapefile) published by Marine Information Research Center.
